# Supplementary material for: Association of α-Dicarbonyls and Advanced Glycation End Products with Insulin Resistance in Non-Diabetic Young Subjects: A Case-Control Study
Source: Nutrients. 2022 Nov 21;14(22):4929. doi: 10.3390/nu14224929 (PMC9695161; doi:10.3390/nu14224929)
Supplement: Supplementary file 1 [file nutrients-14-04929-s001.zip › nutrients-2034986-supplementary.pdf]

Association of α-Dicarbonyls and Advanced Glycation End Products with Insulin Resistance in Non-Diabetic Young Subjects: A Case-Control Study

Melinda Csongová [1], Jean L. J. M. Scheijen [2], Marjo P. H. van de Waarenburg [2], Radana Gurecká [1,3], Ivana Koborová [1], Tamás Tábi [4], Éva Szökö [4], Casper G. Schalkwijk [2] and Katarína Šebeková [1,*]

[1] Institute of Molecular Biomedicine, Medical Faculty, Comenius University, 811 07 Bratislava, Slovakia
[2] Department of Internal Medicine, Maastricht University Medical Centre, 6200 MD Maastricht, The Netherlands
[3] Institute of Medical Physics, Biophysics, Informatics and Telemedicine, Faculty of Medicine, Comenius University, 813 72 Bratislava, Slovakia
[4] Department of Pharmacodynamics, Faculty of Pharmacy, Semmelweis University, 1089 Budapest, Hungary
* Correspondence: kata.sebekova@gmail.com

Supplementary Table S1: Sources of advanced glycation end products determined in the current study

	Non-enzymatic glycation	Reactive α-dicarbonyls			Glycoxidation reactions
		MGO	GO	3-DG	
MG-H1	●	●			
CML	●		●	●	●
CEL	●	●			
Pentosidine	●			●	●

Different metabolic routes lead to the formation of advanced glycation end products (AGEs) of identical structures. All determined AGEs may be formed via nonenzymatic glycation. Methylglyoxal (MGO) modifies arginine residues to form MGO-derived hydroimidazolone-1 (MG-H1) and lysine residues to form N^{ε}-(carboxyethyl)lysine (CEL). 3-deoxyglucosone (3-DG) is a precursor of N^{ε}-(carboxymethyl)lysine (CML) and pentosidine; glyoxal (GO) of CML. CML and pentosidine may be produced via glycoxidation reactions [2–5].

Supplementary Table S2: Correlation between the activity of semicarbazide-sensitive amine oxidase (SSAO) and methylglyoxal (MGO) and MGO-derived advanced glycation end products (AGEs) in males

	**Log MGO**	**Log f-MG-H1**	**f-CEL**	**Log u-MG-H1**	**Log u-CEL**	**Pb-MG-H1**	**Log pb-CEL**
r	-0.056	0.140	0.067	0.156	0.056	-0.005	0.127
p	0.545	0.128	0.451	0.094	0.552	0.957	0.168

f free, *MG-H1* methylglyoxal-derived hydroimidazolone, *CEL* N^{ε}-(carboxyethyl)lysine, *u* urinary, *pb* protein-bound

Supplementary Table S3: Sex-associated alterations in α-dicarbonyls-AGEs-sRAGE axis in insulin resistance

**Glycemia, Fructosamines:** *Sex diff*: ↓ **F vs. M**; *IR effect*: ↑ **IR M; ↑ IR F**<br>**Insulinemia:** *Sex diff*: ↔, ↔; *IR effect*: ↑ **IR M; ↑ IR F**<br>**QUICKI:** *Sex diff*: ↔, ↔; *IR effect*: ↑ **IR M; ↑ IR F**			
**sVAP-1:** *Sex diff*: ↔, ↔;<br>*IR effect*: ↑ **IR F, ↑ IR M**			
**MGO**: *Sex diff*: ↓ **F vs. M**;<br>*IR effect*: ↔, ↔		**GO:** *Sex diff*: ↓ **F vs. M**;<br>*IR effect*: ↑ **IR F •, ↔**	**3-DG:** *Sex diff*: ↓ **F vs. M**;<br>*IR effect*: ↑ **IR F •, ↑ IR M •**
**u-D-lactate:** *Sex diff*: ↑**F vs. M**; *IR effect*: ↔, ↔			
**f-MG-H1:** *Sex diff*: ↓ **F vs. M**;<br>*IR effect*: ↑ **IR F•, ↑ IR M**	**f-CEL:** *Sex diff*: ↓ **F vs. M**;<br>*IR effect*: ↑ **IR F•, ↑ IR M**	**f-CML:** *Sex diff*: ↓ **F vs. M**;<br>*IR effect*: ↔, ↔	
**u-MG-H1:** *Sex diff*: ↑**F vs. M**;<br>*IR effect*: ↑ **IR F•, ↑ IR M**	**u-CEL:** *Sex diff*: ↑**F vs. M**;<br>*IR effect*: ↑ **IR F, ↑ IR M**	**u-CML:** *Sex diff*: ↑**F vs. M**;<br>*IR effect*: ↔, ↔	
**FE$_{MG-H1}$:** *Sex diff*: ↑**F vs. M**;<br>*IR effect*: ↔, ↔	**FE$_{CEL}$:** *Sex diff*: **NS**;<br>*IR effect*: ↔, ↔	**FE$_{CML}$:** *Sex diff*: ↑**F vs. M**;<br>*IR effect*: ↑ **IR F, ↑ IR M**	
**Pb-MG-H1:** Sex diff * IR effect:<br>↑ **IR F vs. IR M, ↓ IR M vs. IS M•**	**Pb-CEL:** *Sex diff*: ↑**F vs. M**;<br>*IR effect*: ↑ **IR F•**, ↔	**Pb-CML:** *Sex diff*: ↑**F vs. M**;<br>*IR effect*: ↔, ↔	***Pb-pent:*** *Sex diff*: ↔, ↔;<br>*IR effect*: ↑ **IR F, ↑ IR M**
cRAGE: *Sex diff*: **NS**; *IR effect*: ↓ **IR F, ↑ IR M•**<br>***esRAGE:*** *Sex diff*: ↓ **F vs. M**; *IR effect*: : ↓ **IR F**, ↔			

IR subjects of both sexes present higher fasting glycemia and insulinemia compared with their IS peers. Higher fructosamine levels corroborate the elevation of glycemia over a longer period. In response to elevated glycemia, sVAP-1 levels increase to stimulate glucose uptake into target tissues [38]. SVAP-1/SSAO converts aminoacetone into MGO [39]. Despite the direct correlation between sVAP-1 protein mass and SSAO activity [40], MGO concentrations did not differ between IS and IR subjects. This corroborates that threonine metabolism is not altered in insulin resistance [41,42]; acetoacetone remains a minor source of MGO unless the supply of threonine is increased. The glyoxalase system rapidly detoxifies MGO into D-lactate [3]. Similar urinary D-lactate excretions in IS and IR subjects indicate that the flux of MGO through the glyoxalase system is not increased in insulin resistance. However, higher serum f-MG-H1 and f-CEL

levels and their higher urinary excretion indirectly point to higher production of MGO in the IR state. Higher GO levels in IR females were not associated with increased f-CML levels or higher renal excretion of CML, probably reflecting that CML is also produced via several other metabolic pathways. Higher fractional excretion of CML corroborates that the kidney regulates the handling of f-AGEs [49]. Among the 3 investigated α-dicarbonyls, only 3-DG was increased in IR subjects; and was accompanied by a mild rise in minor AGE – pentosidine in IR males. While the levels of pb-CML were similar in IS and IR subjects, those of pb-MG-H1 (the dominant pb-AGE) were lower in IR males and pb-CEL concentrations were higher in IR females compared with their IS counterparts. Mechanisms behind these sex differences remain unclear, as protein turnover rate is similar in adolescent males and females [57], and our IR males and females presented with a similar QUICKI. In males, insulin resistance was associated with higher cRAGE levels.

Sex diff. sex differences*, IR* insulin-resistant, *M* males, *F* females, $\uparrow$ increased, $\downarrow$ decreased, $\leftrightarrow$ not affected significantly, • significant predictor of the quantitative insulin sensitivity check index (QUICKI) in the multivariate analysis using the model of the orthogonal projections to latent structures (OPLS), *sVAP-1* soluble vascular adhesion protein-1, *SSAO* semicarbazide-sensitive amino oxidase, *MGO* methylglyoxal, *GO* glyoxal, *3-DG* 3-deoxyglucosone, *f* free, *MG-H1* methylglyoxal-derived hydroimidazolone, CEL N^{ε}-(carboxyethyl)lysine, *CML* N^{ε}-(carboxymethyl)lysine, *pb* protein-bound, *pent* pentosidine, *u* urinary, *FE* fractional excretion, *cRAGE* cleaved soluble receptor for advanced glycation end-products, *esRAGE* endogenous secretory receptor for advanced glycation end-products, *NS* not significant

Supplementary Figure S1: Multivariate regression if independent variables on the quantitative insulin sensitivity check index (QUICKI) using the orthogonal projections to latent structures model in males **(A)** and females **(B)** and plots of variables importance for the projection (VIP) in males **(C)** and females **(D)**

A

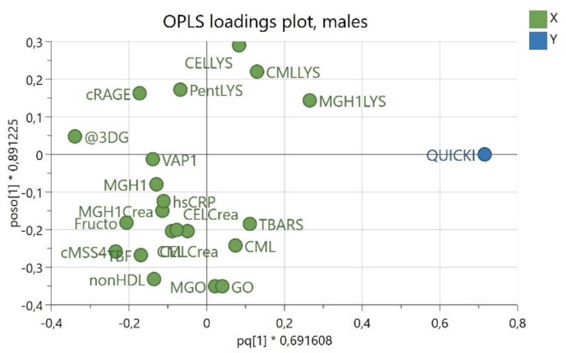

B

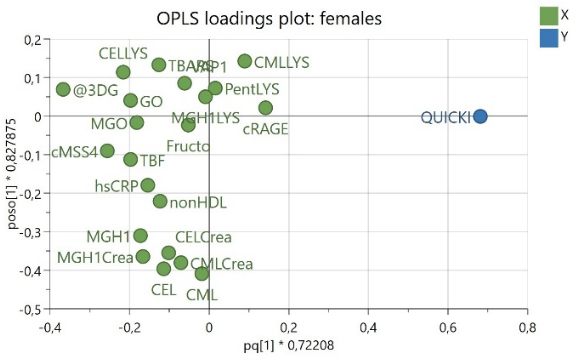

Loading scatter plot displays the relationship between independent x-variables variables (green circles) and the dependent y-variable (QUICKI, blue circle). Independent variables situated in the vicinity of the dependent variable QUICKI show a positive relationship with QUICKI (e.g., MGH1LYS – protein-bound MGH-1 in Fig. 1A), while those on the opposite site far to the left inversely correlate with QUICKI (e.g., @3DG – 3-deoxyglucosone).

C

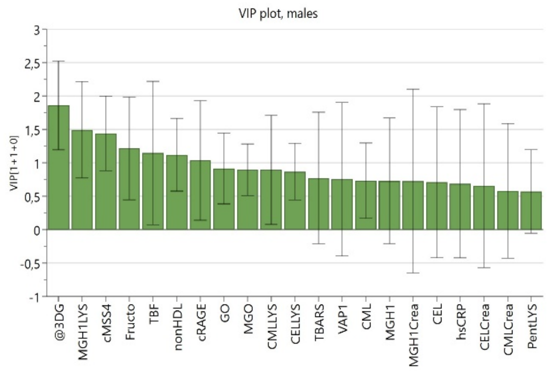

D

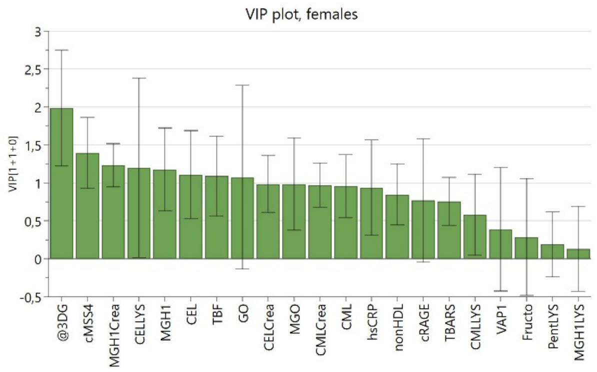

The VIP plot summarizes the importance of the variables to correlate with QUICKI, sorted from high to low, and shows 95% confidence intervals for the VIP. Variables with VIP ≥1.00 are considered significant.

@3DG 3-deoxyglycosone, *MGH1LYS* protein-bound methylglyoxal-derived hydroimidazolone, *cMSS4* continuous metabolic syndrome score without glycemia, *Fructo* fructosamine, *TBF* total body fat, *HDL* high density lipoprotein cholesterol, *cRAGE* cleaved receptor for advanced glycation end products, *GO* glyoxal, *MGO* methylglyoxal, *CMLLYS* protein-bound N^{ε}-(carboxymethyl)lysine, *CELLYS* protein-bound N^{ε}-(carboxyethyl)lysine, *TBARS* thiobarbituric acid reactive substances, *VAP1* soluble vascular adhesion protein-1, *CML* free N^{ε}-(carboxymethyl)lysine, *MGH1* free methylglyoxal-derived hydroimidazolone, *MGH1Crea* urinary methylglyoxal-derived hydroimidazolone/creatinine, *CEL* free N^{ε}-(carboxyethyl)lysine, *hsCRP* high sensitive C reactive protein, *CELCrea* urinary N^{ε}-(carboxyethyl)lysine/creatinine, *CMLCrea* urinary N^{ε}-(carboxymethyl)lysine/creatinine, *PentLYS* protein-bound pentosidine

References

[2] Degenhardt TP, Thorpe SR, Baynes JW. Chemical modification of proteins by methylglyoxal. Cell Mol Biol (Noisy-le-grand). 1998;44:1139-45.
[3] Rabbani N, Xue M, Thornalley PJ. Dicarbonyls and glyoxalase in disease mechanisms and clinical therapeutics. Glycoconj J. 2016;33:513-25.
[4] Dyer DG, Blackledge JA, Thorpe SR, Baynes JW. Formation of pentosidine during nonenzymatic browning of proteins by glucose. Identification of glucose and other carbohydrates as possible precursors of pentosidine in vivo. J Biol Chem. 1991;266:11654-60.
[5] Jono T, Nagai R, Lin X, Ahmed N, Thornalley PJ, Takeya M, et al. Nepsilon-(Carboxymethyl)lysine and 3-DG-imidazolone are major AGE structures in protein modification by 3-deoxyglucosone. J Biochem. 2004;136:351-8.
[38] Li HY, Wei JN, Lin MS, Smith DJ, Vainio J, Lin CH, et al. Serum vascular adhesion protein-1 is increased in acute and chronic hyperglycemia. Clin Chim Acta. 2009;404:149-53.
[39] Deng Y, Yu PH. Assessment of the deamination of aminoacetone, an endogenous substrate for semicarbazide-sensitive amine oxidase. Anal Biochem. 1999;270:97-102.
[40] Koborová I, Gurecká R, Csongová M, Volkovová K, Szökő É, Tábi T, et al. Association between metabolically healthy central obesity in women and levels of soluble receptor for advanced glycation end products, soluble vascular adhesion protein-1, and the activity of semicarbazide-sensitive amine oxidase. Croat Med J. 2017;58:106-16.
[41] Seibert R, Abbasi F, Hantash FM, Caulfield MP, Reaven G, Kim SH. Relationship between insulin resistance and amino acids in women and men. Physiol Rep. 2015;3:e12392.
[42] Vangipurapu J, Stancáková A, Smith U, Kuusisto J, Laakso M. Nine Amino Acids Are Associated With Decreased Insulin Secretion and Elevated Glucose Levels in a 7.4-Year Follow-up Study of 5,181 Finnish Men. Diabetes. 2019;68:1353-8.
[49] Perkins RK, Miranda ER, Karstoft K, Beisswenger PJ, Solomon TPJ, Haus JM. Experimental Hyperglycemia Alters Circulating Concentrations and Renal Clearance of Oxidative and Advanced Glycation End Products in Healthy Obese Humans. Nutrients. 2019;11.
[57] Smith GI, Mittendorfer B. Sexual dimorphism in skeletal muscle protein turnover. J Appl Physiol (1985). 2016;120:674-82.
